# Assessment of Determinants of Paediatric Diarrhoea Case Management Adherence in Pakistan

**DOI:** 10.3390/life13030677

**Published:** 2023-03-02

**Authors:** Asif Khaliq, River Holmes-Stahlman, Danish Ali, Shamshad Karatela, Zohra S. Lassi

**Affiliations:** 1 School of Public Health & Social Work, Queensland University of Technology, Brisbane 4059, Australia; 2 School of Biomedical Sciences, Queensland University of Technology, Brisbane 4059, Australia; 3 Department of Orthodontics, Jinnah Sindh Medical University, Karachi 75510, Pakistan; 4 School of Pharmacy, University of Queensland, Brisbane 4072, Australia; 5 Institute of Tropical Health and Medicine (AITHM), James Cook University, Townsville 4811, Australia; 6 Robinson Research Institute, The University of Adelaide, Adelaide 5005, Australia; 7 School of Public Health, Faculty of Health and Medical Sciences, The University of Adelaide, Adelaide 5005, Australia

**Keywords:** adherence, case management, determinants, diarrhoea, paediatrics

## Abstract

Worldwide, diarrhoea in children under five years of age is the second leading cause of death. Despite having high morbidity and mortality, diarrhoeal diseases can be averted by simple and cost-effective interventions. The Integrated Management of Childhood Illness (IMCI) has proposed the use of Oral Rehydration Salt (ORS) and zinc together with adequate food and fluid intake for the management of acute non-dysenteric watery diarrhoea in children. In the past, few studies examined the determinants of adherence to diarrhoea case management. Therefore, this study measured the determinants of therapeutic and dietary adherence to diarrhoea case management using the third and fourth wave of Pakistan Demographics and Health Surveys (PDHS) datasets. Data from 4068 children between 0 to 59.9 months with positive history of diarrhoea were included, while data on children with dysentery, severe dehydration, and co-morbid condition was excluded. This study reported therapeutic adherence in less than 10% of children in Pakistan, while dietary adherence was reported in 39.2% of children (37.7% in 2012–2013 and 40.7% in 2017–2018). A significant improvement in therapeutic (0.8% in 2012–2013 and 8.1% in 2017–2018) and dietary adherence (37.7% in 2012–2013 and 40.7% in 2017–2018) was reported in the 2017–2018 survey compared to the 2012–2013 survey. In general, children over the age of one year (compared to children <1 year) and of the richer/richest socioeconomic class (compared to poorest/poorer socioeconomic class) showed higher therapeutic and dietary adherence. Therapeutic and dietary adherence among diarrhoeal children can be improved by increasing the awareness and accessibility of ORS, zinc, and essential foods.

## 1. Introduction

Children under five years of age are highly vulnerable to various infections [1]. Pneumonia, diarrhoea, malaria, tuberculosis, and Human Immunodeficiency Virus (HIV) are the five common but preventable infectious diseases known to kill millions of children before their fifth birthday [2,3]. Among various paediatric illnesses, diarrhoea is the second leading cause of death after pneumonia in children below five years [4,5]. The disease is characterised by the passage of three or more stools of a loose consistency on any given day, which leads to fluid and electrolyte loss. If not managed promptly, fluid and electrolyte loss during the diarrhoea results in hypovolemic shock and even death [5,6].

Despite having high mortality associated with diarrhoea, the disease can be averted by simple and cost-effective interventions [7]. To prevent health adversities associated with diarrhoea, it is necessary to break the transmission cycle of the common pathogens causing diarrhoea [5]. This can be executed by providing health education, immunisation with the rotavirus vaccine, exclusive breastfeeding for children under six months, access to safe drinking water, and improved sanitation, such as safe excreta disposal, washing hands with soap, and personal and food hygiene [5,8,9]. However, medical cases of paediatric diarrhoea can be managed by using either oral and/or intravenous fluids [10]. The Integrated Management of Childhood Illness (IMCI) guideline has recommended the use of Oral Rehydration Solution (ORS), zinc, and adequate use of both fluid and food for the management of non-dysenteric diarrhoea with none/some dehydration [7].

ORS was recommended among non-dysenteric diarrhoeal children with none/some dehydration over fifty years ago in 1968 [11]. The ORS is a mixture of glucose and different salts, such as sodium chloride, potassium chloride, and sodium citrate. When mixed with water, this mixture of glucose with different salt can replenish the necessary electrolytes and water loss in faeces during diarrhoea [12]. In addition to ORS, the World Health Organisation (WHO) and UNICEF in 2004 advised using oral zinc because zinc reduces the severity and frequency of diarrhoeal episodes among children, reducing the complications and mortality associated with diarrhoea [11,13]. The global adherence to the guidelines set out by the WHO and UNICEF is still inadequately adhered to on a large scale [7]. In 2012, less than fifty percent of children with diarrhoea received the recommended treatment proposed by the WHO and UNICEF in the IMCI guidelines [7,11,13,14].

The paediatric diarrhoea case management approach in Pakistan is not different from other countries. Most of the studies conducted previously have individually examined the use of ORS and zinc, but a few studies have only assessed the adherence to the co-administration of zinc with ORS. To the best of our knowledge, no studies in Pakistan have thoroughly examined the adherence to the co-administration of zinc with ORS at a large scale. Moreover, adherence to dietary practices has not been investigated in most countries. To investigate the treatment and dietary adherence to the recommended WHO and IMCI guidelines, this study was conducted among children below five years of age using the third and fourth wave of Pakistan Demographics and Health Surveys (PDHS) datasets.

## 2. Methods

### 2.1. Study Design

This study was based on the secondary analysis of the Pakistan Demographic and Health Surveys (PDHS). This study included data from the third and fourth waves of the PDHS conducted in 2012–2013 and 2017–2018. In each PDHS, the data were collected by a team of data collectors, who received adequate training of around three to four weeks related to data collection and interviewing technique. The data collectors in each PDHS interviewed women of reproductive age by means of a survey questionnaire, and there was no predefined exposure and outcome measurement. Due to this, each PDHS has a cross-sectional study design.

### 2.2. Study Population

The study included data from all children aged 0 to 59.9 months with positive diarrhoea history. However, data on all children with dysentery (blood in stool), severe dehydration (received intravenous treatment), and enteric fever (diarrhoea with fever) were excluded. Additionally, data on children simultaneously suffering from diarrhoea with cold, cough, flu, shortness of breath, and malaria, were excluded.

### 2.3. Sample Size and Sampling Methods

The primary sampling unit (PSU) in each Demographic and Health Survey (DHS) were women aged between 15 to 49 years, which were approached via a multistage stratified cluster sampling method for the interview. Initially, the target population of this study was bifurcated into groups based on their place of residence: urban and rural. Enumeration blocks (EBs) were selected randomly from each urban and rural group. In general, each EBs represents a cluster of 200 to 250 households. Finally, from each EB, women were selected following a systematic random method. The National Institute of Population Sciences (NIPS) instructed the data collection team to a select number women from every 28th household for the survey. However, the sample size in each PDHS was calculated using the number of EBs. The list of the EBs in each PDHS was received from the Pakistan Bureau of Statistics and the last census records. During the third wave of PDHS, there were 500 EBs, which increased to 580 during the fourth PDHS wave conducted in 2017–2018. The. The number of EB in 2012–2013 was 500, which increased to 580 in 2017–2018. The survey team multiplied the number of EB with the fixed number “28”, and thus, produced a sample size of 14,000 and 16,240 [15,16].

In this study, children aged below five years with a history of diarrhoea in the last fourteen days were targeted. Data from all the children with dysentery, severe dehydration, enteric fever, and other comorbid condition were excluded. Similarly, data of all the children with normal health status, i.e., no episode of diarrhoea and/or any other common preventable illnesses, i.e., cold, cough, flu, and fever, were also excluded. After excluding the data of ineligible children in this study, we received a total sample size of 5105 (2998 from 2012–2013 and 2107 from 2017–2018).

### 2.4. Study Outcome

The primary outcome of this study is to assess adherence to diarrhoea case management practices in children. The WHO has recommended using ORS, oral zinc sulphate, and adequate fluid and food to manage diarrhoea in children with no and/or some dehydration. The adherence to recommended diarrhoea case management was derived from the consumption of ORS, zinc, adequate fluid, and food intake during the diarrhoeal episodes. In this study, adherence to diarrhoea case management was examined in two steps: (1) therapeutic adherence and (2) dietary adherence. Further information about therapeutic adherence and dietary adherence pertaining to diarrhoea case management is presented in Table 1.

### 2.5. Study Covariates

Different covariates, such as child age (0 to 11 months, 12 to 23 months, 24 to 35 months, 36 to 47 months, and 48 to 59 months), gender (male and female), birth order (first and other), birth type (singlet and twin/triplet), and community factors such as place of residence (urban and rural) were selected. Additionally, maternal factors, such as maternal age (less than 20, 20 to 34 and 35 plus), maternal formal education (none, primary, and secondary/higher), maternal occupation (full-time housewife and working mothers), number of parities (two or less and more than two), and birth in last five years (two or less and more than two), were also included. Similarly, household factors, such as family size (four or less and more than four), number of children under five years (two or less than two and more than two), and wealth index (poorest, poorer, middle, richer, and richest), were also included.

### 2.6. Data Analysis

In this study, two different datasets were obtained from the DHS data repository. After variable selection, the datasets used in this study were merged into a single file. Before formal data analysis initially, preliminary analysis for each variable was performed before formal data analysis to assess the data quality and distribution. Following preliminary analysis, descriptive analysis was carried out. Later, inferential analysis was carried out. The outcome variable of this study was adherence to diarrhoea case management practices, which has two categories: Yes and No. Thus, this study used binomial logistic regression to assess adherence to diarrhoea case management practices with different covariates. Variables with a *p*-value of ≤0.05 and a confidence interval of 95% were considered significant predictors.

### 2.7. Ethical Principles of Study

The data of this study were obtained from the DHS data repository. After obtaining from the DHS data repository, the data were screened thoroughly. The data files were completely devoid of all the information, including any patient identifier, such as participant name, residential address, national identity card number, passport number, and phone number. The ethical principles of anonymity and confidentiality were maintained throughout this research.

## 3. Results

### 3.1. Description of the Study Population

This study analysed data from 4068 children aged 0 months to 59.9 months using datasets of Pakistan Demographic and Health Surveys conducted in 2012–2013 and 2017–2018, respectively. The detail regarding the sociodemographic profile of the study population is presented in Table 1.

More than 40% of children between 0 to 59.9 months received ORS during diarrhoeal illness, but less than a quarter of children received zinc. In 2012–2013, only 2% of the children received zinc, which increased to 12.5% in 2017–2018. Due to this, the 2017–2018 survey reported an increase in treatment adherence to 8.1% from 0.8% in 2012–2013.

Across two survey period, an adequate intake of fluid was reported in around 60% of the children. However, less than half of the children demonstrated an adequate intake of food. Overall, 39.2% of children showed dietary adherence during their diarrhoeal illness (Table 2).

### 3.2. Determinants of Therapeutic Adherence during Diarrhoea Case Management

Our study reported a significant improvement in the therapeutic adherence of paediatric diarrhoea case management across two survey periods. Similarly, zinc intake has improved significantly in the 2017–2018 survey compared with the 2012–2013 survey. However, the intake of ORS did not show any significant change across the two survey periods.

In general, the therapeutic adherence to paediatric diarrhoea case management significantly increased in children aged over 12 months compared to children aged between 0 to 11.9 months. Similarly, the intake of ORS and zinc was significantly higher in children over 12 months compared to children below 12 months.

Compared to children of the poorest socioeconomic class, children of the richest socioeconomic class showed 1.56 (1.01 to 243) higher odds of therapeutic adherence. However, socioeconomic class did not show any association with the use of either ORS or zinc during diarrhoea (Table 3).

Additionally, increased maternal education, increased family size, and decreased number of childbirths in the last five years significantly increased the intake of ORS during diarrhoea.

### 3.3. Determinants of Dietary Adherence during Diarrhoea Case Management

Compared to the 2012–2013 survey, the 2017–2018 survey showed a significant improvement in dietary adherence during diarrhoea case management. Similarly, food intake during the diarrhoea case management increased to 1.26 (1.11 to 1.43) in the 2017–2018 survey compared to the 2012–2013 survey. However, no association was observed between the intake of fluid and survey years.

In general, an improvement in the socioeconomic status from poorest to richest significantly increased the intake of fluid, intake of food, and both (dietary adherence). Similarly, an increase in child age from 12 months to 59.9 significantly increases food intake and dietary adherence by around 1.5- to 2-fold. Additionally, dietary adherence during the diarrhoea case management decreased by 2% (95% CI: 1% to 3%) with increased family size (Table 4).

## 4. Discussion

Diarrhoea is a common preventable paediatric illness that can be managed by simple and cost-effective interventions [17]. Our study examined the adherence to diarrhoea case management practices among paediatric population using datasets of the last two waves of PDHS. To the best of our knowledge, our study is amongst the few studies that measured both therapeutic adherence (coadministration of zinc with ORS) and dietary adherence (adequate fluid intake and food) among diarrhoeal children of Pakistan. Additionally, our study also examined the individual as well as the cumulative adherence of each therapeutic and feeding indicator of diarrhoeal children using nationwide datasets of Pakistan (PDHS datasets). However, other studies from Pakistan had not assessed the therapeutic and dietary adherence exclusively among diarrhoeal children [18,19,20,21].

Our study reported a significant improvement in diarrhoea case management across two survey periods. The 2017–2018 survey showed over ten-fold improvement in therapeutic adherence to diarrhoea case management compared to the former survey of 2012–2013. This increase in therapeutic adherence in 2017–2018 was largely attributed to increased zinc utilisation (Table 3). Despite significant improvement in therapeutic adherence to diarrhoea case management practices, less than 10% of diarrhoeal cases showed therapeutic adherence (Table 2). Researchers from Asian and African regions reported that less than a third of children receive zinc together with ORS [22,23,24,25,26]. However, around 40% of diarrhoeal children received ORS for fluid and electrolyte replenishment [23,24,25,26]. The ORS has ability to reduce diarrhoea associated complications and mortality by replenishing the lost fluids and electrolytes [13], but it fails to alleviate the symptoms of diarrhoea, such as nausea, abdominal pain, and abdominal cramps [27]. Due to this, both the caregiver and care providers opt other methods for the therapeutic management of paediatric diarrhoea, including antimicrobial therapy [27,28].

Across two survey periods, our study demonstrated a remarkable improvement in dietary adherence during diarrhoea case management (Table 3). Our study reported significantly higher dietary adherence among children of the richer/richest socioeconomic classes than children of the poor/poorer socioeconomic classes (Table 3). The low feeding adherence among children of poor/poorer socioeconomic classes is mainly attributed to food insecurity due to poverty. A report by the Pakistan Institute of Development Economics (PIDE) indicated that around one-third of the Pakistani population had been food insecure for over a decade [29]. Poverty is the major cause of food insecurity. Inequalities in food distribution between various members of the household also contribute to low feeding adherence among children of poor/poorer socioeconomic status [30,31]. In general, in most societies, women and children received food at the end, and our study also supported that each member increase in a family reduces the odds of feeding adherence by 2% (1% to 3%), but on the other hand, increases the therapeutic adherence to ORS intake by 2% (1% to 3%). This reflects how the family size and dynamics contribute to diarrhoea case management.

Our study reported significantly higher odds of therapeutic and dietary adherence among children aged 12 to 59.9 months than those aged below one year. The reasons for the low therapeutic and dietary adherence among children aged below one year are unknown. We can postulate that exclusive breastfeeding (EBF) recommendation for children aged below six months can be one of the preponderant reasons for the low adherence among young children. During the EBF period, it is advisable to use ORS, medicine, multivitamins, and vaccines in conjunction with breast milk [32,33]. However, any solid, semi-solid, liquid, and soft food during the first six months of life reflect a deviation from the recommended feeding standards [32].

Moreover, delayed weaning after eight months of age also lowers the odds of adherence among children of this age group [21,33]. Similarly, the cultural practice of food abstinence during diarrhoeal illness and lack of maternal knowledge about diarrhoea case management also contributes to the low adherence [34]. Different studies conducted in Pakistan reflected that most mothers are well aware of zinc and ORS use for managing paediatric diarrhoea [18,35,36]. However, a study conducted in the urban slums of Karachi reported inappropriate diarrhoea case management practices despite having adequate knowledge regarding diarrhoea case management [18]. The knowledge–practice gap for diarrhoea case management can be averted by performing ethnographic studies, and these ethnographic studies would explore the underlying barriers to diarrhoea case management among Pakistani children.

### 4.1. Policy Implications and Recommendations

Despite the significant increase in therapeutic and dietary adherence across two survey periods, this study reported low therapeutic and dietary guidelines of less than 10% and 40%, respectively. Therapeutic and dietary adherence among diarrhoeal cases can be improved by following various prevention and treatment strategies for managing acute paediatric diarrhoea. UNICEF, WHO, and various other national and international bodies have proposed numerous prevention and treatment packages to alleviate the morbidity and mortality associated with diarrhoea. The diarrhoea prevention package includes immunisation against rotavirus and measles, water and sanitation hygiene (WASH), hand hygiene, EBF, and vitamin-A supplementation. However, the treatment package consists of coadministration of zinc and ORS with adequate intake of food and fluid [37]. Our study demonstrated that the therapeutic and dietary indicators for managing acute watery non-dysenteric diarrhoea are not practiced appropriately. In this regard, there is a need to define therapeutic and dietary indicators for the management and control of paediatric diarrhoea. These indicators should be part of national, regional, and institutional surveillance programs [19]. Based on the findings of our study, we proposed a need for standardised dietary practices to manage paediatric diarrhoea among children aged between 6 to 59.9 months [38]. Different papers published in the past proposed using yogurt, banana, and rice with lentils because of their pre-biotic, hyperkalaemic, and antidiarrhoeal properties [18,20,21]. In this regard, promoting the coadministration of zinc and ORS with adequate food and fluid intake during diarrhoea case management is essential.

### 4.2. Strengths and Limitations of the Study

Using the third and fourth waves of the PDHS datasets, this study is among the few studies that measure the therapeutic and dietary adherence of paediatric diarrhoea case management. This study presented paediatric diarrhoea case management at the national level because each PDHS survey’s sample size was calculated from the estimates of the last census [39]. Another more significant strength of this study was the multistage stratified sampling method for selecting participants. Similarly, excluding diarrhoea cases with blood and intravenous therapy from this study clearly defined the study population.

Besides various study strengths, the cross-sectional design of this study weakens the internal validity of this study. The data collectors in each PDHS survey relied on the mother’s response regarding the diarrhoea prevalence during the last fourteen days. Similarly, the information related to diarrhoea case management was also subjective. There might be high chances of reporting bias, recall bias, or both. Moreover, the food and fluid intake questions were very general, which asked about the amount of food and/or fluid given to a child during diarrhoeal episodes. Hence, this study did not assess dietary adherence properly.

## 5. Conclusions

Adherence to diarrhoea case management is a pivotal tool for reducing the morbidity and mortality associated with paediatric diarrhoea. The WHO and UNICEF have proposed using ORS, zinc, and adequate food and fluid intake for the proper case management of paediatric diarrhoea. Worldwide, millions of children die from lack of adherence, although Pakistan showed a significant improvement in therapeutic and dietary adherence between the two survey periods (from 2012–2013 to 2017–2018). Still, less than half of the children under five years old are adherent to the recommended diarrhoea case management guidelines. A set of therapeutic strategies, such as coadministration of zinc and ORS with adequate intake of food and fluid, have the potential to avert the therapeutic and dietary non-adherence among diarrhoeal children.

## Figures and Tables

**Table 1:** Operational definition of therapeutic and dietary adherence pertaining to paediatric diarrhoea case management.

	Adherence	Non-Adherence
Therapeutic	Coadministration of ORS and zinc for the management of acute watery non-dysenteric diarrhoea with none/some dehydration. OR Children who received either home solution and/or antibiotic pills and/or antibiotic syrup and/or antimotility drug and/or any unknown pill and/or non-antibiotic injection and/or rice starch and/or mint extract and/or herbal remedy with both ORS and zinc were also considered adherent to diarrhoea case management.	The therapeutic non-adherence may encompass treatment with any of the following medicine/remedies either without ORS or zinc or both: (1) Home solution (2) Antibiotic pills (3) Antibiotic syrup (4) Antimotility drug (5) Antibiotic injection (6) Intravenous fluid (7) Any unknown pill (8) Non-antibiotic injection (9) Unknown injection (10) Rice starch (11) Mint extract (12) Herbal remedy (13) Other treatment (14) No treatment.
Dietary adherence	Adequate intake ^1^ of both food and fluid during the diarrhoeal illness.	Inadequate intake ^2^ of either fluid or food or both during the diarrheoal illness.

^1^ Adequate intake of food and fluid = either equal or greater intake of food/fluid during the diarrhoeal illness than does the routine food/fluid intake. ^2^ Inadequate intake of food and fluid = the intake of food/fluid during the diarrhoeal illness should be lower than does the routine food/fluid intake. Food abstinence and fluid restrictions are examples of inadequate fluid/food intake.

**Table 2:** Sociodemographic features of the study population.

Variable	2012–2013 (n = 2002)	2017–2018 (n = 2066)	Total (n = 4068)
*Child factors*			
***Child age (categorical)***			
0 to 11.9 months 12 to 23.9 months 24 to 35.9 months 36 to 47.9 months 48 to 59.9 months	506 (12.4%) 554 (13.6%) 415 (10.2%) 312 (7.7%) 215 (5.3%)	577 (14.2%) 567 (13.9%) 423 (10.4%) 289 (7.1%) 210 (5.2%)	1083 (26.6%) 1121 (27.6%) 838 (20.6%) 601 (14.8%) 425 (10.4%)
***Child sex***			
Male Female	1065 (26.2%) 937 (23.0%)	1093 (26.9%) 973 (23.9%)	2158 (53%) 1910 (47%)
***Birth order***	*3.47 ± 2.73*		*3.36 ± 2.28*
***Birth order (categorical)***			
Primipara Multipara	471 (23.5%) 1531 (76.5%)	523 (25.3%) 1543 (74.7%)	994 (24.5%) 3074 (75.5%)
***Birth type***			
Singlet Twin/triplet	1958 (977.8%) 44 (2.2%)	2016 (97.5%) 50 (2.5%)	3974 (97.7%) 94 (2.3%)
***Maternal factors***			
***Maternal age***			
15 to 19 years 20 to 24 years 25 to 29 years 30 to 34 years 35 to 39 years 40 to 44 years 45 to 49 years	68 (3.4%) 490 (24.5%) 626 (31.3%) 454 (22.7%) 256 (12.8%) 86 (4.3%) 22 (1.1%)	86 (4.2%) 479 (23.2%) 660 (31.9%) 468 (22.7%) 275 (13.3%) 77 (3.7%) 21 (1%)	154 (3.8%) 969 (23.8%) 1286 (31.6%) 922 (22.7%) 531 (13.1%) 163(4.0%) 43 (1.1%)
***Maternal formal education***			
None Primary Secondary Higher	1116 (55.7%) 302 (15.1%) 380 (19.0%) 204 (10.2%)	1007 (48.7%) 318 (15.4%) 456 (22.1%) 285 (13.8%)	2123 (52.2%) 620 (15.2%) 836 (20.6%) 489 (12.0%)
***Maternal work***			
Full-time housewife Working mothers	1602 (76.9%) 400 (23.1%)	1857 (89.9%) 209 (10.1%)	3459 (85%) 609 (15%)
***Number of parities***	3.73 ± 2.38	3.50 ± 2.20	3.61 ± 2.30
***Birth in last 5 years***	1.80 ± 0.74	1.74 ± 0.71	1.77 ± 0.72
***Household factors***			
***Family size***	9.43 ± 5.02	9.52 ± 5.04	9.47 ± 5.03
***Wealth index***			
Poorest Poorer Middle Richer Richest	446 (22.3%) 443 (22.1%) 392 (19.6%) 400 (20.0%) 321 (16.0%)	432 (21.7%) 482 (23.3%) 448 (20.9%) 366 (17.7%) 338 (16.4%)	894 (22%) 925 (22.7%) 824 (20.3%) 766 (18.8%) 659 (16.2%)
***Treatment factors***			
***Use of ORS***			
No Yes	1148 (57.3%) 854 (42.7%)	1213 (58.7%) 853 (41.3%)	2361 (58%) 1707 (42%)
***Use of zinc***			
No Yes	1962 (98%) 40 (2%)	1807 (87.4%) 259 (12.5%)	4028 (92.7%) 299 (7.3%)
***Treatment adherence***			
No adherence Adherence	1984 (99.2%) 18 (0.8%)	1882 (93.9%) 184 (8.1%)	3866 (95%) 202 (5%)
***Use of fluid intake***			
None or less than usual More or same as usual	821 (41%) 1181 (59%)	818 (39.5%) 1248 (60.5%)	1639 (40.3%) 2429 (59.7%)
***Food and diet intake***			
None or less than usual More or same as usual	1175 (58.6%) 956 (41.3%)	1110 (53.7%) 956 (42.3%)	2285 (56.2%) 1783 (43.8%)
***Dietary adherence***			
No adherence Adherence	1247 (62.3%) 755 (37.7%)	1225 (59.3%) 841 (40.7%)	2472 (60.8%) 1596 (39.2%)
***Community factors***			
***Place of residence***			
Urban Rural	829 (41.4%) 1173 (58.6%)	917 (44.3%) 1149 (55.7%)	1746 (42.9%) 2322 (57.1%)

**Table 3:** Assessing the determinants of diarrhoea treatment adherence in children below five years of age.

Variable	Intake of ORS ^1^		Intake of Zinc ^2^		Treatment Adherence ^3α^	
	Unadjusted Odd (95% CI)	Adjusted Odds (95% CI)	Unadjusted Odd (95% CI)	Adjusted Odds (95% CI)	Unadjusted Odd (95% CI)	Adjusted Odds (95% CI)
***Year*** *2012–2013* *2017–2018*	Ref 0.94 (0.83 to 1.07)		Ref 7.03 (50.1 to 9.86) *	Ref 7.14 (5.08 to 10.03) *	Ref 10.77 (6.61 to 17.55) *	Ref 10.97 (6.73 to 17.89) *
***Child age (categorical)*** *0 to 11.9 months* *12 to 23.9 months* *24 to 35.9 months* *36 to 47.9 months* *48 to 59.9 months*	Ref 1.94 (1.63 to 2.31) * 1.74 (1.44 to 2.09) * 1.57 (1.27 to 1.92) * 1.29 (1.02 to 1.63) *	Ref 1.95 (1.64 to 2.32) * 1.78 (1.47 to 2.15) * 1.58 (1.28 to 1.94) * 1.31 (1.04 to 1.66) *	Ref 1.69 (1.21 to 2.36) * 1.48 (1.03 to 2.14) * 1.02 (0.70 to 1.63) 1.98 (1.31 to 3.01) *	Ref 1.80 (1.28 to 2.54) * 1.57 (1.08 to 2.28) * 1.19 (0.76 to 1.84) 2.18 (1.43 to 3.32) *	Ref 1.96 (1.28 to 2.98) * 1.87 (1.19 to 2.93) * 1.33 (0.79 to 2.26) 2.17 (1.30 to 3.63) *	Ref 2.06 (1.34 to 3.17) * 2.02 (1.34 to 3.17) * 1.49 (0.87 to 2.56) 2.43 (1.44 to 4.12) *
***Child sex*** *Male* *Female*	Ref 0.97 (0.85 to 1.09)		Ref 1.10 (0.86 to 1.39)		Ref 1.00 (0.75 to 1.33)	
***Birth order***	0.98 (0.96 to 1.01)		0.97 (0.92 to 1.02)		0.98 (0.92 to 1.05)	
***Birth order (categorical)*** *Primipara* *Multipara*	Ref 0.95 (0.82 to 1.10)		Ref 0.94 (0.72 to 1.23)		Ref 0.92 (0.67 to 1.28)	
***Birth type*** *Singlet* *Twin/triplet*	Ref 0.81 (0.53 to 1.24)		Ref 0.41 (0.12 to 1.30)		Ref 0.65 (0.19 to 1.99)	
***Maternal age*** *15 to 19 years* *20 to 24 years* *25 to 29 years* *30 to 34 years* *35 to 39 years* *40 to 44 years* *45 to 49 years*	Ref 1.23 (0.86 to 1.76) 1.30 (0.91 to 1.84) 1.48 (1.03 to 2.11) * 1.53 (1.06 to 2.23) * 1.19 (0.75 to 1.89) 1.46 (0.73 to 2.91)		Ref 0.91 (0.47 to 1.71) 0.84 (0.45 to 1.59) 0.95 (0.50 to 1.81) 1.14 (0.59 to 2.22) 1.19 (0.54 to 2.65) 0.28 (0.03 to 2.23)		Ref 1.09 (0.48 to 2.46) 0.93 (0.41 to 2.09) 1.10 (0.48 to 2.48) 1.39 (0.60 to 3.21) 1.66 (0.63 to 4.35) 0.50 (0.05 to 4.17)	
***Maternal formal education*** *None* *Primary* *Secondary* *Higher*	Ref 0.98 (0.81 to 1.18) 1.44 (1.23 to 1.70) * 1.41 (1.16 to 1.72) *	Ref 0.95 (0.79 to 1.15) 1.36 (1.15 to 1.61) * 1.28 (1.03 to 1.58) *	Ref 1.28 (0.91 to 1.79) 1.25 (0.92 to 1.69) 1.55 (1.09 to 2.19) *		Ref 1.18 (0.78 to 1.81) 1.25 (0.86 to 1.81) 1.84 (1.24 to 2.74) *	
***Maternal work*** *Full-time housewife* *Working mothers*	Ref 0.89 (0.75 to 1.07)		Ref 0.73 (0.51 to 1.06)		Ref 0.90 (0.61 to 1.36)	
***Number of parities***	0.98 (0.95 to 1.01)		0.97 (0.92 to 1.02)		0.98 (0.92 to 1.05)	
***Birth in last 5 years***	0.87 (0.80 to 0.95) *	0.89 (0.81 to 0.97) *	0.91 (0.77 to 1.07)		0.83 (0.68 to 1.02)	
***Family size***	1.01 (1.00 to 1.02) *	1.02 (1.01 to 1.03) *	1.00 (0.97 to 1.02)		0.99 (0.96 to 1.02)	
***Wealth index*** *Poorest* *Poorer* *Middle* *Richer* *Richest*	Ref 1.18 (0.97 to 1.43) 1.35 (1.11 to 1.64) * 1.32 (1.08 to 1.61) * 1.74 (1.41 to 2.13) *		Ref 1.05 (0.73 to 1.51) 1.07 (0.73 to 1.55) 1.01 (0.68 to 1.48) 1.46 (1.01 to 2.12) *		Ref 1.03 (0.67 to 1.59) 1.01 (0.64 to 1.57) 0.79 (0.49 to 1.29) 1.55 (1.01 to 2.39) *	Ref 1.01 (0.64 to 1.55) 0.95 (0.60 to 1.51) 0.84 (0.51 to 1.38) 1.56 (1.01 to 2.43) *
***Place of residence*** *Urban* *Rural*	Ref 0.71 (0.63 to 0.81) *	Ref 0.79 (0.69 to 0.91) *	Ref 0.86 (0.68 to 1.10)		Ref 0.89 (0.67 to 1.18)	

* = A significant association with various study covariates having *p*-value ≤ 0.05, ^1^ = Odds of ORS intake was adjusted with child age, maternal formal education, birth in last five years, family size, and place of residence, ^2^ = Odds of zinc intake was adjusted with year of survey and child age, ^3^ = Odds of treatment adherence was adjusted with year of survey, child age, and wealth index, α = treatment adherence is the sum of adherence to the intake of both ORS and zinc during the diarrhoeal illness.

**Table 4:** Dietary adherence during diarrhoea case management among children below five years of age.

**Variable**	**Adequate Fluid Intake**		**Adequate Food Intake**		**Dietary Adherence**	
	**Unadjusted Odd** **(95% CI)**	**Adjusted Odds ^1^** **(95% CI)**	**Unadjusted Odd** **(95% CI)**	**Adjusted Odds ^2^** **(95% CI)**	**Unadjusted Odd** **(95% CI)**	**Adjusted Odds ^3^**^α^ **(95% CI)**
***Year*** *2012–2013* *2017–2018*	Ref 1.06 (0.93 to 1.20)		Ref 1.22 (1.08 to 1.35) *	Ref 1.26 (1.11 to 1.43) *	Ref 1.13 (1.01 to 1.28) *	Ref 1.16 (1.02 to 1.32) *
***Child age (categorical)*** *0 to 11.9 months* *12 to 23.9 months* *24 to 35.9 months* *36 to 47.9 months* *48 to 59.9 months*	Ref 0.91 (0.76 to 1.08) 0.89 (0.74 to 1.07) 0.86 (0.71 to 1.06) 0.79 (0.63 to 1.00)		Ref 1.83 (1.54 to 2.18) * 1.74 (1.44 to 2.09) * 2.15 (1.75 to 2.63) * 2.011 (1.60 to 2.52) *	Ref 1.86 (1.56 to 2.21) * 1.78 (1.48 to 2.15) * 2.22 (1.81 to 2.73) * 2.06 (1.63 to 2.58) *	Ref 1.71 (1.41 to 2.07) * 1.77 (1.41 to 2.07) * 2.00 (1.62 to 2.46) * 2.01 (1.59 to 2.53) *	Ref 1.78 (1.49 to 2.13) * 1.74 (1.44 to 2.11) * 2.06 (1.67 to 2.54) * 2.04 (1.61 to 2.57) *
***Child sex*** *Male* *Female*	Ref 0.94 (0.83 to 1.07)		Ref 1.05 (0.93 to 1.19)		Ref 1.01 (0.89 to 1.14)	
***Birth order***	0.95 (0.93 to 0.98) *		0.97 (0.94 to1.001)		0.97 (0.94 to 0.99) *	
***Birth order (categorical)*** *Primipara* *Multipara*	Ref 0.92 (0.79 to 1.07)		Ref 0.98 (0.85 to 1.14)		Ref 0.98 (0.85 to 1.14)	
***Birth type*** *Singlet* *Twin/triplet*	Ref 0.99 (0.65 to 1.51)		Ref 0.82 (0.54 to 1.29)		Ref 0.75 (0.49 to 1.17)	
***Maternal age*** *15 to 19 years* *20 to 24 years* *25 to 29 years* *30 to 34 years* *35 to 39 years* *40 to 44 years* *45 to 49 years*	Ref 1.08 (0.76 to 1.53) 1.19 (0.85 to 1.68) 1.08 (0.76 to 1.53) 0.94 (0.65 to 1.35) 1.04 (0.66 to 1.64) 0.52 (2.65 to 1.04)		Ref 1.08 (0.76 to 1.53) 1.40 (0.99 to 1.97) 1.38 (097 to 1.96) 1.18 (0.82 to 1.71) 1.15 (0.73 to 1.81) 1.05 (0.52 to 2.10)		Ref 1.27 (0.88 to 1.83) 1.61 (1.12 to 2.31) * 1.54 (1.07 to 2.22) * 1.27 (0.86 to 1.86) 1.42 (0.89 to 2.27) 1.06 (0.52 to 2.19)	
***Maternal formal education*** *None* *Primary* *Secondary* *Higher*	Ref 1.44 (1.19 to 1.73) * 1.27 (1.08 to 1.50) * 1.33 (1.08 to 1.63) *		Ref 1.31 (1.10 to 1.57) * 1.31 (1.11 to 1.54) * 1.29 (1.06 to 1.58) *		Ref 1.38 (1.15 to 1.65) * 1.31 (1.12 to 1.55) * 1.37 (1.12 to 1.67) *	
***Maternal work*** *Full-time housewife* *Working mothers*	Ref 0.87 (0.73 to 1.04)		Ref 0.86 (0.72 to 1.02)		Ref 0.91 (0.75 to 1.08)	
***Number of parities***	0.95 (0.93 to 0.98) *		0.98 (0.95 to 1.01)		0.98 (0.95 to 1.01)	
***Birth in last 5 years***	0.99 (0.91 to 1.08)		0.98 (0.91 to 1.07)		1.00 (0.91 to 1.09)	
***Family size***	0.99 (0.97to 1.00)		0.99 (0.98 to 1.01)		0.98 (0.97 to 1.00)	0.98 (0.97 to 0.99) *
***Wealth index*** *Poorest* *Poorer* *Middle* *Richer* *Richest*	Ref 1.29 (1.07 to 1.55) * 1.48 (1.22 to 1.80) * 1.85 (1.51 to 1.82) * 1.48 (1.21 to 1.82) *	Ref 1.29 (1.07 to 1.55) * 1.48 (1.22 to 1.80) * 1.85 (1.51 to 2.26) * 1.48 (1.21 to 1.82) *	Ref 1.29 (1.01 to 1.47) * 1.23 (1.02 to 1.49) * 1.54 (1.27 to 1.88) * 1.44 (1.17 to 1.76) *	Ref 1.23 (1.02 to 1.49) * 1.24 (1.02 to 1.51) * 1.61 (1.31 to 1.96) * 1.47 (1.20 to 1.81) *	Ref 1.21 (0.99 to 1.46) 1.24 (1.02 to 1.51) * 1.66 (1.36 to 2.03) * 1.47 (1.20 to 1.82) *	Ref 1.23 (1.01 to 1.49) * 1.26 (1.03 to 1.54) * 1.73 (1.41 to 2.11) * 1.53 (1.24 to 1.90) *
***Place of residence*** *Urban* *Rural*	Ref 0.91 (0.81 to 1.04)		Ref 0.91 (0.80 to 1.03)		Ref 0.87 (0.77 to 0.99) *	

* = A significant association with various study covariates having *p*-value ≤ 0.05, ^1^ = Odds of fluid intake was adjusted with wealth index, ^2^ = Odds of food intake was adjusted with year of survey, child age, and wealth index, ^3^ = Odds of dietary adherence was adjusted with year of survey, child age, family size, and wealth index, α = dietary adherence is the sum of adequate intake of both fluid and food during the diarrhoeal illness.

## Data Availability

The data from this study can be retrieved from the DHS program (www.dhsprogram.com (accessed on 23 September 2022)).
